# Efficacy of First Line Systemic Chemotherapy and Multikinase Inhibitors in Advanced Hepatocellular Carcinoma: A Systematic Review and Network Meta-Analysis

**DOI:** 10.3389/fonc.2021.654020

**Published:** 2021-03-31

**Authors:** Songporn Oranratnachai, Sasivimol Rattanasiri, Anantaporn Pooprasert, Amarit Tansawet, Thanyanan Reungwetwattana, John Attia, Ammarin Thakkinstian

**Affiliations:** ^1^ Department of Clinical Epidemiology and Biostatistics, Faculty of Medicine Ramathibodi Hospital, Mahidol University, Bangkok, Thailand; ^2^ Oncology Clinic, Sriphat Medical Center, Faculty of Medicine, Chiang Mai University, Chiang Mai, Thailand; ^3^ Oncology Unit, Division of Internal Medicine, Uttaradit Hospital, Uttaradit, Thailand; ^4^ Department of Surgery, Faculty of Medicine Vajira Hospital, Navamindradhiraj University, Bangkok, Thailand; ^5^ Division of Medical Oncology, Department of Medicine, Faculty of Medicine Ramathibodi Hospital, Mahidol University, Bangkok, Thailand; ^6^ Centre for Clinical Epidemiology and Biostatistics, School of Medicine and Public Health, Hunter Medical Research Institute, University of Newcastle, New Lambton, NSW, Australia

**Keywords:** chemotherapy, first-line systemic treatment, hepatocellular carcinoma, multikinase inhibitors, network meta-analysis

## Abstract

**Background:**

Hepatocellular carcinoma (HCC) is the third most fatal cancer, with a 5-year survival rate of 18%. Standard frontline-therapy is multikinase inhibitors (MKIs), but accessibility is still limited, particularly in developing countries. This network meta-analysis (NMA) aimed to compare the efficacy of usual chemotherapy vs MKIs.

**Method:**

Randomised-controlled trials (RCTs) comparing any among chemotherapy vs MKIs in treatment-naïve patients with advanced HCCs were identified from MEDLINE and SCOPUS databases. Overall survival (OS) and progression-free survival (PFS) probabilities and times were extracted from Kaplan-Meier curves using Digitizer, and then converted to individual patient time-to-event data. A one-stage mixed-effect survival model was applied to estimate median OS and PFS. A two-stage NMA was applied for the overall response rate and adverse events (AEs) outcome.

**Results:**

A total of 20 RCTs were eligible for NMA. Lenvatinib was the best treatment among single MKIs, with median OS and PFS of 9 and 6.3 months, without significant differences in AEs relative to other MKIs. Median OS and PFS were 0.70 (-0.42, 1.83) and 2.17 (1.41, 2.93) months longer with Lenvatinib than Sorafenib. Among chemotherapy agents, FOLFOX4 had the longest median OS and PFS at 7.9 and 4.3 months, respectively, without significant AEs compared to other chemotherapies. The combination of Sorafenib+Doxorubicin prolonged median OS and PFS to 12.7 and 6.3 months, respectively.

**Conclusion:**

Use of the MKIs Lenvatinib or Sorafenib as first line systemic treatment for advanced HCC could be beneficial. However, FOLFOX4 might be the optimal choice in a developing country where the health-care budget is limited.

## Introduction

Hepatocellular carcinoma (HCC) is the third most common cause of cancer-related death worldwide (1). The common risk factors are chronic hepatitis B or C virus infection and cirrhosis from any cause. Treatment of HCC depends on the disease stage, which simultaneously considers liver function, performance status, and tumor burden (2, 3). Early-stage disease usually requires only local treatment, whereas advanced-stage disease may need Multikinase Inhibitors (MKIs) given preserved liver functions; otherwise, supportive care is the only option.

Sorafenib was the first MKI approved by the Food and Drug Administration (FDA) in 2017 as frontline therapy for advanced-stage HCC. Sorafenib increased median overall survival (OS) to 10.7 months compared with 7.9 months in placebo (4). Other MKIs [i.e., Brivanib (5), Sunitinib (6), and Linifanib (7)] were subsequently tested in phase-III trials, but failed to improve OS relative to Sorafenib, until Lenvatinib, approved by the FDA in 2018, was shown to prolong OS to 13.6 months (8).

To date, there are nine systematic reviews (SRs) of frontline treatment options for advanced/unresectable HCC published between 2012-2018 (9–17). Most SRs used direct meta-analysis to compare efficacy between chemotherapy agents or between MKIs, but none compared efficacy of chemotherapy and MKIs (10–17). Of those SRs, only one used network meta-analysis (NMA) (9), including 6 randomised-controlled trials (RCTs) with 4,812 patients, to indirectly compare the efficacy and drug toxicity between 5 MKIs (i.e., Sorafenib, Brivanib, Sunitinib, Linifanib, and Sorafenib+Erlotinib) and placebo. Although MKIs might improve clinical outcomes, the accessibility of these drugs is limited due to high cost. Therefore, we aimed to compare the efficacy and adverse events (AEs) of chemotherapy and MKIs on OS, and progression-free survival (PFS) using a NMA approach.

## Methods

This SR and NMA were conducted according to Preferred Reporting Items for Systematic Reviews and Meta-Analyses (PRISMA) and registered to PROSPERO (CRD42019145620). Relevant studies were identified from MEDLINE *via* PubMed and SCOPUS databases through to 30^th^ November, 2019. The search terms were constructed based on patients (advanced or unresectable HCC), interventions (i.e., chemotherapy and MKIs), and outcomes of interest (see **Table 1**). Titles and abstracts were screened by one reviewer (SO) then randomly checked by second reviewer (SR). The full texts were then independently selected by two reviewers (SO and SR), and disagreements were resolved through discussion with a third reviewer (ATh). The most recent studies were selected when there were multiple publications. The reference lists were reviewed to identify additional relevant studies.

**Table 1:** Search terms.

Domain	Search terms for MEDLINE	Search terms for SCOPUS
**Patients**	(Hepatoma OR “liver cell cancer” OR “liver cancer”) AND (advance OR advanced OR unresectable OR unresected OR inoperable OR metastasis)	(“hepatocellular carcinoma” OR hepatoma OR “liver cell cancer” OR “liver cancer”) AND (advance OR advanced OR unresectable OR unresected OR inoperable OR metastasis).
**Interventions**	(“chemotherapy” OR adriamycin OR oxaliplatin OR fluorouracil OR xeloda OR nolatrexed) OR (“targeted therapy” OR “multikinase inhibitors” OR nexavar OR lenvima OR sutent OR brivanib OR linifanib)	(chemotherapy OR adriamycin OR doxorubicin OR oxaliplatin OR fluorouracil OR xeloda OR capecitabine OR nolatrexed) OR (“targeted therapy” OR “multikinase inhibitors” OR nexavar OR sorafenib OR lenvima OR lenvatinib OR sutent OR sunitinib OR brivanib OR linifanib).
**Outcome**	Survival OR (toxicity OR toxicities OR “adverse event” OR “adverse events” OR drug toxicity) OR response	Survival OR (toxicities OR “adverse events”) OR response
**Filter/** **Limit to**	clinical study, clinical trial, comparative study, controlled clinical trial, randomized controlled trial, observational study	article, reviews, conference paper

Search terms between each domain will be combined with ‘AND’ Boolean.

RCTs were eligible if they met the following criteria: studies in adults with advanced HCC who were treatment-naïve; comparing any pair of chemotherapy agents (e.g., Doxorubicin, Oxaliplatin, Fluorouracil or Capecitabine, Nolatrexed), MKIs (e.g., Sorafenib, Lenvatinib, Brivanib, Sunitinib, Linifanib), or placebo; and had at least one of the following outcomes: OS, PFS, overall response rate (ORR), and AEs.

RCTs were excluded if they compared systemic with non-medical treatments (e.g., surgery, liver transplant), local treatments (e.g., chemoembolisation, radiotherapy, and hepatic arterial/portal vein chemo infusion) or non-chemotherapy/MKI (e.g., hormonal treatment, tumor vaccine, or gene therapy). If there were in an untranslatable languages, or had insufficient data for pooling after three contact attempts with authors they were also excluded.

### Interventions

Interventions of interest were placebo, chemotherapy (e.g., Doxorubicin, Nolatrexed, combination of Cisplatin, Interferon α-2b, Doxorubicin, and Fluorouracil (PIAF), combination of Fluorouracil, Leucovorin, and Oxaliplatin (FOLFOX4)), monotherapy of MKIs (e.g., Sorafenib, Sunitinib, Brivanib, Linifanib, Dovitinib, Lenvatinib, and Nintedanib), combine chemotherapy with MKIs (e.g., Sorafenib+Doxorubicin, Sorafenib+combination of Gemcitabine and Oxaliplatin (GEMOX)), and combination of MKIs (e.g. Sorafenib+Erlotinib, Sorafenib+Everolimus, and Bevacizumab+Erlotinib).

### Outcome of Interests

Outcome of interest were OS, PFS, ORR, and AEs. OS was defined as time since randomization to death from any cause. PFS was defined as time since randomization to first occurrence of disease progression or death from cancer-related event. If RCTs (4, 5, 7, 18–21) reported only time to progression which was defined as time since randomization to disease progression, time to progression was used instead of PFS. ORR was defined as the proportion of patients who had a best objective tumor response of complete response or partial response. Tumor response was classified using World Health Organisation (WHO) (22) or the response evaluation criteria in solid tumors (RECIST) (23) criteria according to RCTs’ report. AEs were at least one of grade 3 or higher of following AEs as for the National Cancer Institute’s Common Terminology Criteria for Adverse Events (24): anemia, neutropenia, thrombocytopenia, diarrhea, hand-foot skin reaction, hypertension, or hyponatremia. If RCTs reported these individual AEs rather than overall AEs, the AE with the maximum incidence was used.

### Data Extraction and Quality Assessment

Two reviewers (SO and AP) independently extracted the following data: general characteristics (i.e., number of patients, RCT phase, country), patient characteristics (i.e., gender, mean age, percent hepatitis B/C, Child-Pugh A classification, Barcelona Clinic Liver Cancer stage C, portal vein involvement, extrahepatic spreading), treatment regimens, and outcomes (i.e., complete response and partial response, OS, PFS, and AEs).

The probabilities and times of OS and PFS were also extracted from the Kaplan-Meier curve using Digitizer program (25), and then used to simulate individual patient time-to-event data (26). Any disagreements between the two reviewers were resolved by consensus with a third reviewer (ATh). The main outcome of interest was OS; and secondary outcomes were PFS, ORR, and AEs.

Two reviewers (SO and ATa) used the Revised Cochrane Risk of Bias tool (RoB 2) (27) to assess the quality of RCTs; and any disagreements were resolved by consensus with a third reviewer (Ath).

### Statistical Analysis

Interventions were coded according to treatment as placebo, single-chemotherapy, combined-chemotherapy, combined-chemotherapy with MKIs, single-MKIs, and combined-MKIs. For individual patient time-to-event data for OS and PFS, a one-stage approach using a mixed-effect parametric survival model (28) was applied to obtain relative treatment effects. Appropriate survival distributions (e.g., Weibull, exponential, log-logistic, log-normal, generalized gamma) were assessed using Akaike’s Information Criterion (AIC); and the model with the smallest AIC was used.

For dichotomous outcomes (i.e., ORR and AEs), the relative treatment effect, risk ratio (RR), were compared using a two-stage NMA. The probability of being the best treatment in maximizing ORR and minimal AEs was assessed using a rankogram and the Surface Under the Cumulative Ranking curve (SUCRA). Transitivity was explored by comparing the distribution of co-variables (e.g., gender, co-morbidity, Child-Pugh classification, major vascular invasion, extrahepatic spreading) among comparisons. A comparison-adjusted funnel plot was used to assess publication bias.

All analyses were performed with STATA version 16.0 (29). A two-sided p-value less than 0.05 was considered statistically significant for all tests.

## Results

### Characteristics of Included Studies

**Figure 1:**
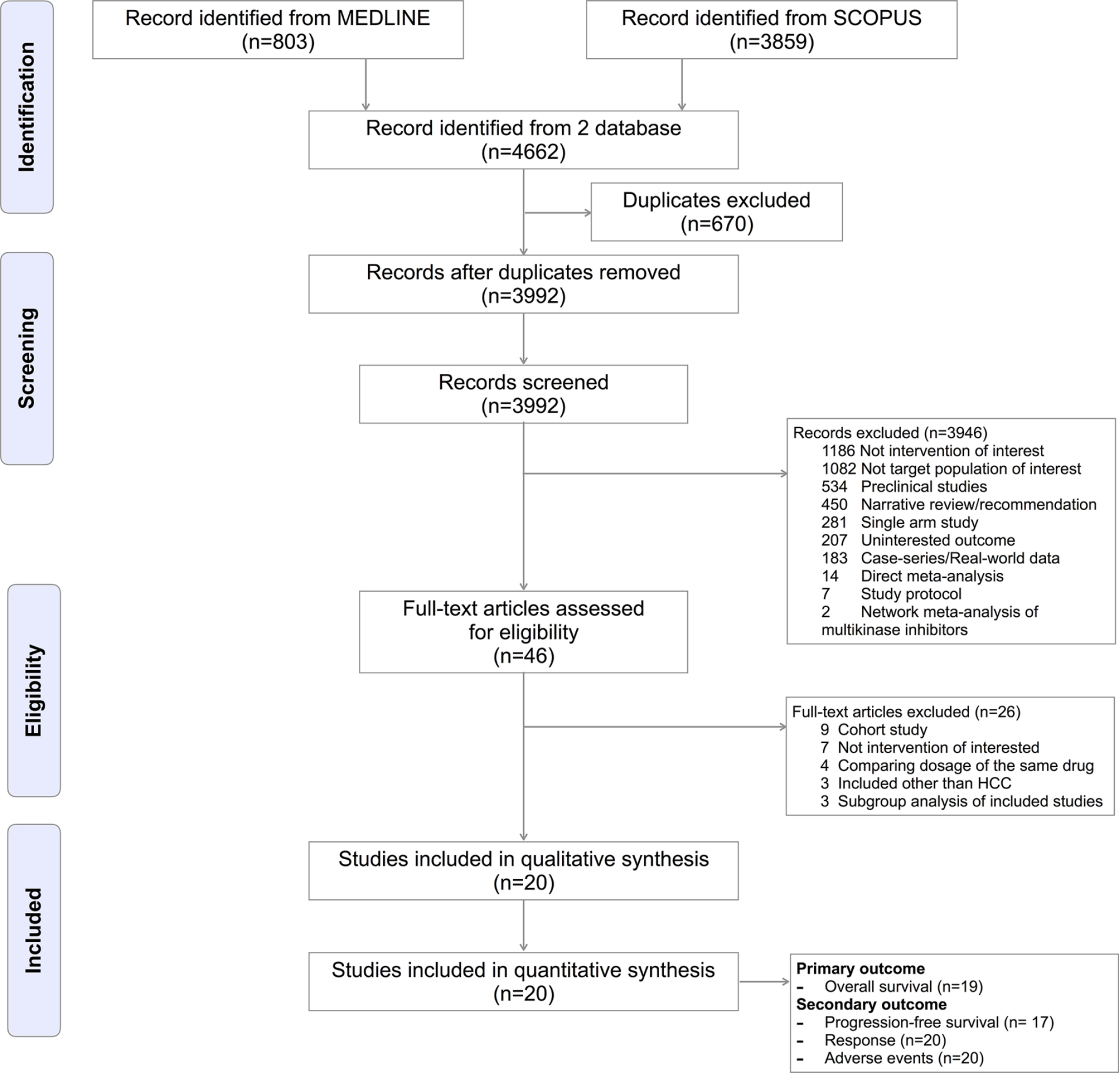
PRISMA flow diagram.

**Figure 2:**
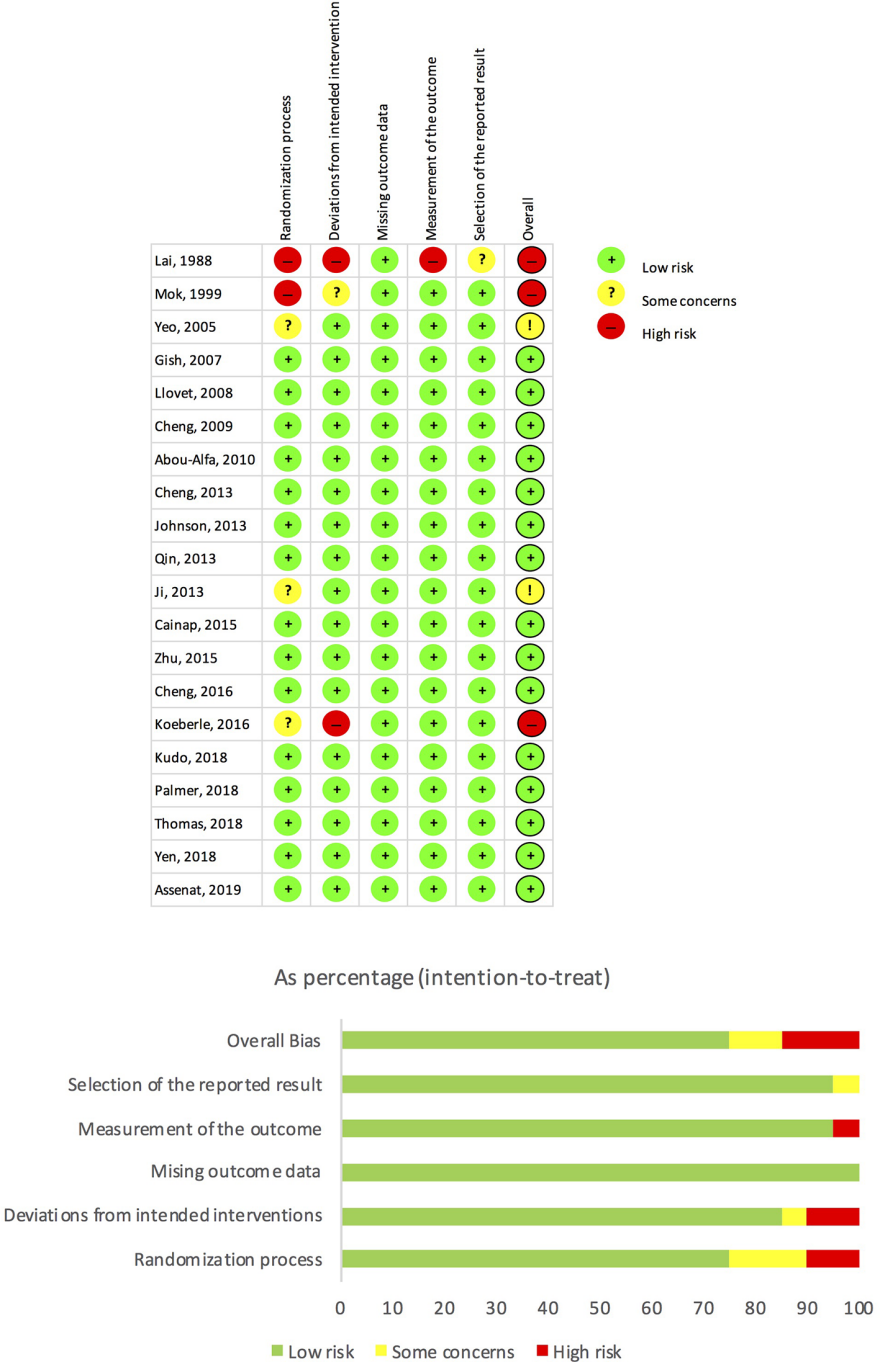
Risk of bias assessment.

A total of 4,662 studies were identified; and reasons for exclusion were reported in the PRISMA flow diagram see **Figure 1**. The overall risk of bias was low at 75%; see **Figure 2**. Inter-observer agreement between reviewers was high for both data extraction (kappa = 0.96) and risk of bias assessment (kappa = 0.88).

**Figure 3:**
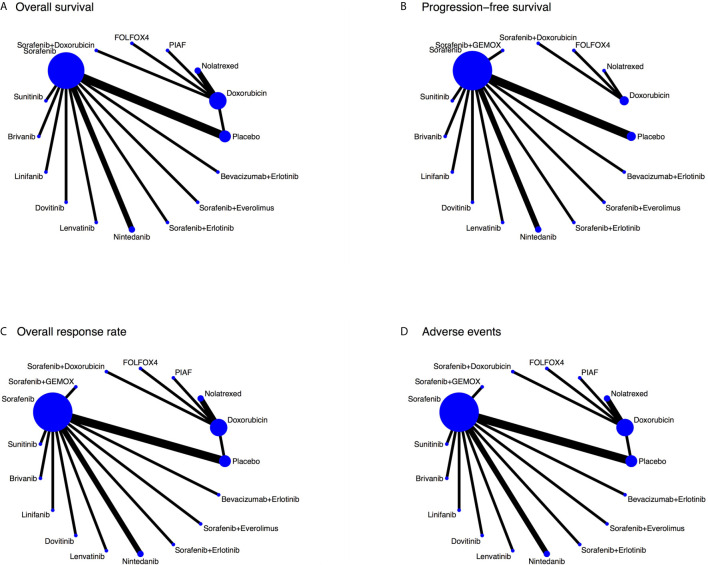
Network map for each outcome. (**A** – overall survival, **B** – progression-free survival, **C** – overall response rate, and **D** – adverse events).

Twenty RCTs with 7,846 patients were included in the NMA (4–8, 18, 19, 21, 30–40). Most were conducted in Asia (40%), in male patients (76.0% - 91.5%), with Child-Pugh A disease except for one study focusing on Child-Pugh B disease (33). The mean age was 49.3 to 65.6 years. A network map for each outcome was constructed see **Figure 3**, and the characteristics of the included RCTs are shown in more detail in **Table 2**.

**Table 2:** Characteristics of included studies.

Author, Year	Treatment	n	RCT Phase	Region	%Male	Mean age	%HBV	%HCV	%Child A	%BCLC C	%PVI	%EHS	Outcomes
Lai (34)	Doxorubicin vs no treatment	106	3	AP	86.8	51.4	73.6	–	–	–	–	–	OS, ORR, AEs
Mok (35)	Nolatrexed vs Doxorubicin	54	2	AP	90.7	54.3	77.8	5.6	–	–	48.1	31.5	OS, ORR, AEs
Yeo et al. (40)	PIAF vs Doxorubicin	188	3	–	91.5	49.3	81.0	6.0	85.1	–	48.5	–	OS, ORR, AEs
Gish (32)	Nolatrexed vs Doxorubicin	445	3	US Eu Af	–	–	22.2	39.3	73.9	–	–	–	OS, PFS, ORR, AEs
Llovet (4)	Sorafenib vs placebo	602	3	US Eu AusNZ	87.0	65.6	18.4	28.1	96.5	82.4	38.4	51.3	OS, PFS, ORR, AEs
Cheng (18)	Sorafenib vs placebo	226	3	AP	85.4	52.5	73.0	8.4	97.3	95.6	35.4	68.6	OS, PFS, ORR, AEs
Abou-Alfa et al. (30)	Sorafenib+Doxorubicin vs Doxorubicin	96	2	US Eu AP	76.0	62.6	10.4	17.7	97.9	–	30.2	58.3	OS, PFS, ORR, AEs
Cheng et al. (6)	Sunitinib vs Sorafenib	1074	3	AP	83.3	55.1	53.8	21.6	99.6	85.3	32.1	–	OS, PFS, ORR, AEs
Johnson et al. (5)	Brivanib vs Sorafenib	1155	3	US Eu AP AusNZ	83.7	57.8	44.3	20.3	92.0	77.3	19.3	49.7	OS, PFS, ORR, AEs
Qin et al. (37)	FOLFOX4 vs Doxorubicin	371	3	AP	88.7	49.4	91.4	6.7	87.9	80.1	–	58.2	OS, PFS, ORR, AEs
Ji et al. (33)	Sorafenib vs no treatment	189	3	AP	84.1	59.0	84.1	2.6	0.0	87.8	–	–	OS, PFS, ORR, AEs
Cainap et al. (7)	Linifanib vs Sorafenib	1035	3	US Eu AP AusNZ	85.0	56.6	53.2	25.0	94.4	82.2	43.4	58.3	OS, PFS, ORR, AEs
Zhu et al. (19)	Sorafenib+Erlotinib vs Sorafenib	720	3	US Eu AP	80.7	–	35.4	26.5	97.4	85.0	40.4	58.9	OS, PFS, ORR, AEs
Cheng et al. (20)	Dovitinib vs Sorafenib	165	2	AP	84.8	55.5	–	–	99.4	97.6	–	–	OS, PFS, ORR, AEs
Koeberle et al. (21)	Sorafenib+Everolimus vs Sorafenib	105	2	–	83.8	62.2	17.1	28.6	82.9	72.4	30.5	55.2	OS, PFS, ORR, AEs
Kudo et al. (8)	Lenvatinib vs Sorafenib	954	3	US Eu AP	84.0	58.0	50.0	23.0	99.0	79.0	21.0	61.0	OS, PFS, ORR, AEs
Palmer et al. (36)	Nintedanib vs Sorafenib	93	2	Eu	79.6	61.5	11.8	22.6	98.9	73.1	33.3	65.6	OS, PFS, ORR, AEs
Thomas et al. (38)	Bevacizumab+Erlotinib vs Sorafenib	90	2	US	–	61.8	–	–	85.6	66.7	21.1	33.3	OS, PFS, ORR, AEs
Yen et al. (39)	Nintedanib vs Sorafenib	95	2	AP	87.4	58.5	63.2	15.8	99.0	87.4	42.1	68.4	OS, PFS, ORR, AEs
Assenat et al. (31)	Sorafenib+GEMOX vs Sorafenib	83	2	Eu	89.2	62.0	3.6	15.7	–	85.5	26.5	68.7	PFS, ORR, AEs

AEs, adverse events; Af, Africa; AP, Asia-Pacific; AusNZ, Australia and New Zealand; BCLC C, Barcelona Clinic Liver Cancer stage C; Child A, Child Pugh Classification A; EHS, extrahepatic spreading; Eu, Europe; FOLFOX4, fluorouracil, leucovorin, and oxaliplatin; GEMOX, gemcitabine, oxaliplatin; HBV, hepatitis B viral infection; HCV, hepatitis C viral infection; ORR, overall response rate; OS, overall survival; PFS, progression-free survival; PIAF, cisplatin, interferon α-2b, doxorubicin, and fluorouracil; PVI, portal vein involvement; RCT, Randomised-controlled Trial; US, United States.

### Overall Survival

Nineteen RCTs (4–8, 18, 19, 21, 30, 32–40) assessed OS with 16 treatment regimens, see **Figure 3A**. Sorafenib+GEMOX was excluded due to the Kaplan-Meier curve not being reported. One-stage NMA with a mixed-effect accelerated failure time model with log-normal distribution see **Table 3**, and 5000-replication bootstrap was applied to estimate the median OS of each treatment, see **Table 4**. Among single-chemotherapy regimens, the median OS of Doxorubicin was a month longer than Nolatrexed, but this was not significant (6.9 versus 5.9 months), whereas the median OS of PIAF and FOLFOX4 were not much different (i.e., 7.3 versus 7.9 months). Median OS of single-MKI regimens varied from 6.7 to 9.0 months, in which Lenvatinib/Nintedanib and Sunitinib had the longest and shortest median OSs respectively. Combined-MKI regimens showed some additional benefit with Sorafenib+Doxorubicin having a median OS of 12.7 months. All relative treatment effects by median OS were then compared among active treatments and placebo/no-treatment see **Table 5**. None of the chemotherapy agents was statistically significantly different when compared to placebo, with differences in median OS ranging from -0.2 to 1.8 months. However, six of seven single-MKIs were significantly different to placebo, prolonging median OS by 0.6 to 3.0 months. Among combination regimens, Sorafenib+Erlotinib and Sorafenib+Doxorubicin, showed median OS (95% confidence interval (CI)) longer than placebo by 2.55 (1.04, 4.05) and 6.62 (0.92, 12.33) months, respectively. Comparing single-MKIs to each other, Sorafenib and Lenvatinib improved median OS by 1.62 (0.80, 2.44) and 2.32 (0.93, 3.71) months, respectively compared to Sunitinib.

**Table 3:** Mixed effect parametric survival models using different distributions of survival time.

Outcome	Survival distribution	Exponential	Weibull	Log-normal	Log-logistic	Gamma
**OS**	**Log likelihood**	-19415.874	-19177.68	-18883.637	-18951.691	-19090.474
	**AIC**	38865.75	38391.36	37803.27	37939.38	38216.95
**PFS**	**Log likelihood**	-15103.572	-14908.65	-14205.728	-14318.184	-14742.737
	**AIC**	30241.14	29853.3	28447.46	28672.37	29521.47

AIC, Akaike’s information criterion, OS, overall survival, PFS, progression-free survival.

**Table 4:** Estimations of median overall survival and median progression-free survival for each treatment regimen.

Treatment regimen	Median OS (95% CI)(months)	Median PFS (95%CI)(months)
Placebo	6.08 (5.44, 6.72)	3.00 (2.70, 3.29)
Doxorubicin	6.91 (5.96, 7.85)	3.27 (2.98, 3.55)
Nolatrexed	5.88 (4.67, 7.08)	3.20 (2.75, 3.66)
PIAF	7.25 (4.90, 9.60)	–
FOLFOX4	7.89 (6.22, 9.57)	4.30 (3.65, 4.96)
Sorafenib + Doxorubicin	12.70 (7.08, 18.32)	6.27 (4.13, 8.40)
Sorafenib + GEMOX	–	4.57 (3.08, 6.05)
Sorafenib	8.32 (7.74, 8.89)	4.09 (3.88, 4.29)
Sunitinib	6.70 (5.79, 7.61)	3.83 (3.40, 4.26)
Brivanib	7.64 (6.61, 8.68)	4.40 (3.88, 4.91)
Linifanib	7.81 (6.70, 8.93)	5.05 (4.40, 5.70)
Dovitinib	8.44 (6.08, 10.80)	3.57 (2.67, 4.48)
Lenvatinib	9.02 (7.77, 10.27)	6.26 (5.48, 7.05)
Nintedanib	9.03 (6.75, 11.33)	3.50 (2.63, 4.36)
Sorafenib + Erlotinib	8.63 (7.23, 10.02)	3.56 (3.07, 4.05)
Sorafenib + Everolimus	9.37 (6.07, 12.66)	4.72 (3.16, 6.28)
Bevacizumab + Erlotinib	7.31 (4.72, 9.90)	5.52 (3.63, 7.41)

CI, Confidence Interval; FOLFOX4, fluorouracil, leucovorin, and oxaliplatin; GEMOX, gemcitabine, oxaliplatin; OS, overall survival; PFS, progression-free survival; PIAF, cisplatin, interferon α-2b, doxorubicin, and fluorouracil.

**Table 5:** Comparisons of median overall survival among treatment regimens.

**Placebo**	0.83 (-0.35, 2.01)	-0.20 (-1.62, 1.22)	1.18 (-1.31, 3.67)	1.81 (-0.02, 3.65)	6.62 (0.92, 12.33)	2.24 (1.46, 3.03)	0.62 (0.92, 12.33)	1.57 (0.39, 2,75)	1.74 (0.48, 2.99)	2.37 (-0.11, 4.84)	2.94 (1.56, 4.32)	2.96 (0.50, 5.41)	2.55 (1.04, 4.05)	3.29 (-0.11, 6.69)	1.24 (-1.46, 3.94)
	**Doxorubicin**	-1.03 (-2.14, 0.08)	0.35 (-1.93, 2.62)	0.98 (-0.40, 2.37)	5.79 (0.23, 11.35)	1.41 (0.07, 2.75)	-0.21 (-1.70, 1.29)	0.74 (-0.85, 2.32)	0.91 (-0.72, 2.53)	1.53 (-1.14, 4.21)	2.11 (0.35, 3.87)	2.13 (-0.54, 4.79)	1.72 (-0.13, 3.58)	2.46 (-1.11, 6.02)	0.41 (-2.45, 3.26)
		**Nolatrexed**	1.38 (-1.14, 3.90)	2.01 (0.23, 3.80)	6.82 (1.13, 12.51)	2.44 (0.92, 3.96)	0.82 (-0.85, 2.49)	1.77 (0.02, 3.51)	1.93 (0.16, 3.71)	2.56 (-0.20, 5.33)	3.14 (1.25, 5.04)	3.16 (0.43, 5.89)	2.75 (0.76, 4.74)	3.49 (-0.14, 7.12)	1.44 (-1.53, 4.40)
			**PIAF**	0.64 (-2.02, 3.30)	5.44 (-0.58, 11.47)	1.06 (-1.49, 3.62)	-0.55 (-3.18, 2.07)	0.39 (-2.27, 3.05)	0.56 (-2.15, 3.27)	1.19 (-2.29, 4.66)	1.77 (-1.05, 4.59)	1.78 (-1.64, 5.21)	1.37 (-1.48, 4.23)	2.11 (-2.06, 6.28)	0.06 (-3.53, 3.65)
				**FOLFOX4**	4.81 (-0.94, 10.55)	0.43 (-1.52, 2.37)	-1.19 (-3.25, 0.87)	-0.25 (-2.38, 1.88)	-0.08 (-2.23, 2.08)	0.55 (-2.47, 3.58)	1.13 (-1.11, 3.37)	1.14 (-1.88, 4.17)	0.74 (-1.59, 3.06)	1.47 (-2.36, 5.31)	-0.58 (-3.77, 2.61)
					**Sorafenib+** **Doxorubicin**	-4.38 (-10.13, 1.38)	-6.00 (-11.76, -0.24)	-5.05 (-10.87, 0.76)	-4.88 (-10.70, 0.94)	-4.25 (-10.47, 1.96)	-3.68 (-9.53, 2.18)	-3.66 (-9.91, 2.58)	-4.07 (-10.00, 1.86)	-3.33 (-9.92, 3.25)	-5.38 (-11.65, 0.88)
						**Sorafenib**	-1.62 (-2.44, -0.80)	-0.67 (-1.60, 0.25)	-0.51 (-1.53, 0.52)	0.12 (-2.27, 2.52)	0.70 (-0.42, 1.83)	0.72 (-1.65, 3.09)	0.31 (-0.99, 1.61)	1.05 (-2.26, 4.36)	-1.00 (-3.64, 1.63)
							**Sunitinib**	0.94 (-0.28, 2.17)	1.11 (-0.16, 2.39)	1.74 (-0.78, 4.27)	2.32 (0.93, 3.71)	2.34 (-0.14, 4.82)	1.93 (0.41, 3.45)	2.67 (-0.72, 6.05)	0.62 (-2.11, 3.34)
								**Brivanib**	0.17 (-1.19, 1.53)	0.80 (-1.74, 3.34)	1.38 (-0.76, 2.83)	1.39 (-1.14, 3.92)	0.98 (-0.60, 2.57)	1.72 (-1.68, 5.13)	-0.33 (-3.11, 2.45)
									**Linifanib**	0.63 (-1.98, 3.24)	1.21 (-0.32, 2.74)	1.22 (-1.36, 3.80)	0.82 (-0.84, 2.47)	1.55 (-1.90, 5.01)	-0.50 (-3.33, 2.33)
										**Dovitinib**	0.58 (-2.06, 3.21)	0.59 (-2.75, 3.93)	0.19 (-2.54, 2.91)	0.92 (-3.15, 5.00)	-1.13 (-4.65, 2.39)
											**Lenvatinib**	0.01 (-2.61, 2.63)	-0.39 (-2.12, 1.33)	0.34 (-3.15, 3.84)	-1.71 (-4.58, 1.17)
												**Nintedanib**	-0.41 (-3.10, 2.29)	0.33 (-3.75, 4.41)	-1.72 (-5.23, 1.78)
													**Sorafenib+** **Erlotinib**	0.74 (-2.81, 4.28)	-1.31 (-4.26, 1.64)
														**Sorafenib+** **Everolimus**	-2.05 (-6.22, 2.12)
															**Bevacizumab** **+Erlotinib**

Values in cell are difference of median overall survival along with 95% confidence interval.

FOLFOX4, fluorouracil, leucovorin, and oxaliplatin; PIAF, cisplatin, interferon α-2b, doxorubicin, and fluorouracil.

Considering current common treatments (i.e., Doxorubicin, FOLFOX4, Sorafenib, and Lenvatinib), Lenvatinib showed the longest OS followed by Sorafenib, FOLFOX4, and Doxorubicin with median OS times of 9.0, 8.3, 7.9, and 6.9 months, respectively. Lenvatinib and Sorafenib showed significantly longer OSs than Doxorubicin. Among chemotherapy agents, FOLFOX4 had the longest OS, and was statistically significant when compared to Nolatrexed (2.01 months, 95% CI of 0.23, 3.80).

### Progression-Free Survival

Seventeen RCTs (4–8, 18, 19, 21, 30–33, 36–39) with 16 regimens were included in the NMA of PFS, see **Figure 3B**. Median PFS varied from 3.0 to 6.3 months, see **Table 4**. Comparisons of median PFS indicated all regimens had significantly longer PFSs compared with placebo, except Doxorubicin, Nolatrexed, Dovitinib, Nintedanib, and Sorafenib+Erlotinib, see **Table 6**. Among chemotherapy agents, only FOLFOX4 had 1.31 (0.58, 2.03) months significantly longer PFS than placebo. Among single-MKIs, Lenvatinib had significantly prolonged PFS of 1.2 to 2.8 months when compared to other agents. Also, Lenvatinib could significantly prolong PFS by 2.0 to 3.1 months when compared with other chemotherapy agents.

**Table 6:** Comparisons of median progression-free survival between treatment regimens.

**Placebo**	0.27 (-0.15, 0.68)	0.21 (-0.34, 0.76)	1.31 (0.58, 2.03)	3.27 (1.12, 5.41)	1.57 (0.03, 3.11)	1.09 (0.77, 1.41)	0.83 (0.33, 1.34)	1.40 (0.82, 1.98)	2.05 (1.35, 2.75)	0.58 (-0.40, 1.55)	3.26 (2.43, 4.09)	0.50 (-0.45, 1.45)	0.57 (0.00, 1.13)	1.72 (0.09, 3.36)	2.53 (0.60, 4.45)
	**Doxorubicin**	-0.06 (-0.51, 0.38)	1.04 (0.39, 1.68)	3.00 (0.78, 5.22)	1.30 (-0.21, 2.81)	0.82 (0.46, 1.18)	0.56 (0.05, 1.08)	1.13 (0.53, 1.73)	1.78 (1.07, 2.49)	0.31 (-0.63, 1.25)	2.99 (2.15, 3.83)	0.23 (-0.67, 1.13)	0.30 (-0.28, 0.87)	1.45 (-0.12, 3.03)	2.26 (0.34, 4.17)
		**Nolatrexed**	1.10 (0.31, 1.89)	3.06 (0.79, 5.34)	1.36 (-0.17, 2.90)	0.88 (0.38, 1.39)	0.63 (0.00, 1.26)	1.19 (0.50, 1.89)	1.85 (1.05, 2.64)	0.37 (-0.63, 1.37)	3.06 (2.14, 3.97)	0.29 (-0.67, 1.25)	0.36 (-0.30, 1.02)	1.52 (-0.09, 3.12)	2.32 (0.35, 4.29)
			**FOLFOX4**	1.96 (-0.38, 4.30)	0.26 (-1.36, 1.89)	-0.22 (-0.90, 0.47)	-0.47 (-1.25, 0.31)	0.09 (-0.74, 0.93)	0.75 (-0.17, 1.67)	-0.73 (-1.84, 0.38)	1.96 (0.93, 2.98)	-0.81 (-1.87, 0.25)	-0.74 (-1.57, 0.08)	0.42 (-1.27, 2.10)	1.22 (-0.77, 3.21)
				**Sorafenib+** **Doxorubicin**	-1.70 (-4.28, 0.89)	-2.18 (-4.32, -0.04)	-2.43 (-4.61, -0.26)	-1.87 (-4.06, 0.33)	-1.22 (-3.47, 1.04)	-2.69 (-5.02, -0.36)	-0.01 (-2.28, 2.27)	-2.77 (-5.08, -0.46)	-2.70 (-4.88, -0.53)	-1.55 (-4.23, 1.13)	-0.74 (-3.59, 2.10)
					**Sorafenib** **+GEMOX**	-0.48 (-1.99, 1.03)	-0.74 (-2.30, 0.83)	-0.17 (-1.76, 1.42)	0.48 (-1.16, 2.12)	-0.99 (-2.75, 0.77)	1.69 (-0.01, 3.39)	-1.07 (-2.82, 0.68)	-1.01 (-2.58, 0.57)	0.15 (-2.02, 2.32)	0.95 (-1.51, 3.42)
						**Sorafenib**	-0.26 (-0.66, 0.14)	0.31 (-0.18, 0.80)	0.96 (0.34, 1.59)	-0.51 (-1.45, 0.43)	2.17 (1.41, 2.93)	-0.59 (-1.53, 0.35)	-0.53 (-1.01, -0.04)	0.63 (-0.97, 2.24)	1.43 (-0.48, 3.35)
							**Sunitinib**	0.57 (-0.07, 1.20)	1.22 (0.48, 1.96)	-0.26 (-1.27, 0.76)	2.43 (1.57, 3.29)	-0.34 (-1.34, 0.67)	-0.27 (-0.77, 2.54)	0.89 (-0.77, 2.54)	1.69 (-0.27, 3.65)
								**Brivanib**	0.65 (-0.14, 1.45)	-0.82 (-1.89, 0.25)	1.86 (0.97, 2.76)	-0.90 (-1.97, 0.17)	-0.83 (-1.52, -0.15)	0.32 (-1.36, 2.00)	1.12 (-0.86, 3.11)
									**Linifanib**	-1.48 (-2.61, -0.34)	1.21 (0.23, 2.19)	-1.55 (-2.68, -0.43)	-1.49 (-2.28, -0.70)	-0.33 (-2.06, 1.40)	0.47 (-1.55, 2.49)
										**Dovitinib**	2.69 (1.48, 3.89)	-0.08 (-1.37, 1.21)	-0.01 (-1.05, 1.03)	1.14 (-0.68, 2.97)	1.95 (-0.21, 4.10)
											**Lenvatinib**	-2.76 (-3.99, -1.54)	-2.70 (-3.61, -1.79)	-1.54 (-3.32, 0.24)	-0.74 (-2.83, 1.35)
												**Nintedanib**	0.07 (-0.97, 1.10)	1.22 (-0.60, 3.04)	2.03 (-0.11, 4.17)
													**Sorafenib+** **Erlotinib**	1.16 (-0.51, 2.82)	1.96 (-0.02, 3.94)
														**Sorafenib+** **Everolimus**	0.80 (-1.73, 3.34)
															**Bevacizumab** **+Erlotinib**

Values are differences of median progression-free survival along with 95% confidence interval.

FOLFOX4, fluorouracil, leucovorin, and oxaliplatin; GEMOX, gemcitabine, oxaliplatin.

Among combined-regimens, Sorafenib+Doxorubicin, Sorafenib+GEMOX, Sorafenib+Everolimus, and Bevacizumab+Erlotinib had median PFS 3.27 (1.12, 5.41), 1.57 (0.03, 3.11), 1.72 (0.09, 3.36), and 2.53 (0.60, 4.45) months significantly longer than placebo.

### Overall Response Rate

Twenty RCTs (4–8, 18, 19, 21, 30–40), with 17 regimens were included to estimate RRs (95% CI) of ORR see **Figure 3C** and **Table 7**. Among chemotherapy agents, FOLFOX4 showed 3.05 (1.13, 8.22) and 8.93 (1.52, 52.59) times significantly higher ORR than Doxorubicin and Nolatrexed respectively. PIAF demonstrated 5.84 (1.13, 30.25) times significantly higher ORR than Nolatrexed. Among MKIs, Lenvatinib, Linifanib, and Brivanib showed 9.04 (2.63, 31.07), 5.15 (1.48, 17.90), and 4.18 (1.23, 14.17) times significantly higher ORR than placebo, whereas the other MKIs were not significant. Also, Lenvatinib was significantly superior to Sorafenib, Sunitinib, Brivanib, Dovitinib, and Nintedanib with RRs of 2.89 (1.96, 4.26), 2.66 (1.45, 4.85), 2.16 (1.29, 3.64), 5.14 (1.68, 15.74), and 4.59 (1.77, 11.93), respectively. Furthermore, Lenvatinib showed 1.71 (0.80, 3.61) and 1.81 (0.53, 6.12) times higher ORR than Sorafenib+Erlotinib and Bevacizumab+Erlotinib, but lower ORR than Sorafenib+Everolimus, FOLFOX4, and PIAF, although none of these was significant. The SUCRAs indicated that the highest ranked treatments were Sorafenib+Everolimus followed by FOLFOX4 (**Table 7**).

**Table 7:** Estimations of relative treatment effects of overall response rate.

**Placebo** **6.2 (0)**	8.48 (0.48,149.47)	2.89 (0.12,72.77)	16.91 (0.87,327.25)	25.84 (1.24,538.24)	13.25 (0.59,297.40)	5.29 (1.00,28.10)	3.13 (0.97,10.09)	3.40 (0.97,11.99)	4.18 (1.23,14.17)	5.15 (1.48,17.90)	1.76 (0.36,8.48)	9.04 (2.63,31.07)	1.97 (0.46,8.49)	5.30 (1.39,20.18)	31.84 (1.46,694.55)	5.01 (0.96,25.98)
0.12 (0.01,2.08)	**Doxorubicin** **56.2 (0)**	0.34 (0.08,1.49)	2.00 (0.96,4.17)	3.05 (1.13,8.22)	1.56 (0.47,5.19)	0.62 (0.02,17.28)	0.37 (0.02,8.19)	0.40 (0.02,9.22)	0.49 (0.02,11.15)	0.61 (0.03,13.88)	0.21 (0.01,5.47)	1.07 (0.05,24.26)	0.23 (0.01,5.82)	0.63 (0.03,14.83)	3.76 (0.06,253.49)	0.59 (0.02,16.16)
0.35 (0.01,8.69)	2.93 (0.67,12.74)	**Nolatrexed** **33.4 (0.3)**	5.84 (1.13,30.25)	8.93 (1.52,52.59)	4.58 (0.69,30.55)	1.83 (0.05,69.04)	1.08 (0.03,33.39)	1.18 (0.04,37.49)	1.44 (0.05,45.37)	1.78 (0.06,56.45)	0.61 (0.02,21.97)	3.12 (0.10,98.67)	0.68 (0.02,23.46)	1.83 (0.06,60.09)	11.00 (0.13,952.42)	1.73 (0.05,64.63)
0.06 (0.00,1.14)	0.50 (0.24,1.05)	0.17 (0.03,0.89)	**PIAF** **75.2 (7.6)**	1.53 (0.44,5.25)	0.78 (0.19,3.20)	0.31 (0.01,9.39)	0.18 (0.01,4.47)	0.20 (0.01,5.04)	0.25 (0.01,6.09)	0.30 (0.01,7.58)	0.10 (0.00,2.98)	0.53 (0.02,13.24)	0.12 (0.00,3.17)	0.31 (0.01,8.09)	1.88 (0.03,135.44)	0.30 (0.01,8.78)
0.04 (0.00,0.81)	0.33 (0.12,0.88)	0.11 (0.02,0.66)	0.65 (0.19,2.25)	**FOLFOX4** **83.8 (30.4)**	0.51 (0.11,2.43)	0.20 (0.01,6.55)	0.12 (0.00,3.14)	0.13 (0.00,3.53)	0.16 (0.01,4.26)	0.20 (0.01,5.31)	0.07 (0.00,2.08)	0.35 (0.01,9.28)	0.08 (0.00,2.22)	0.21 (0.01,5.66)	1.23 (0.02,93.29)	0.19 (0.01,6.13)
0.08 (0.00,1.69)	0.64 (0.19,2.12)	0.22 (0.03,1.46)	1.28 (0.31,5.22)	1.95 (0.41,9.25)	**Sorafenib+** **Doxorubicin** **68.8 (8)**	0.40 (0.01,13.63)	0.24 (0.01,6.55)	0.26 (0.01,7.36)	0.32 (0.01,8.91)	0.39 (0.01,11.09)	0.13 (0.00,4.33)	0.68 (0.02,19.38)	0.15 (0.00,4.62)	0.40 (0.01,11.82)	2.40 (0.03,191.69)	0.38 (0.01,12.76)
0.19 (0.04,1.00)	1.60 (0.06,44.32)	0.55 (0.01,20.65)	3.20 (0.11,95.84)	4.88 (0.15,156.18)	2.50 (0.07,85.52)	**Sorafenib** **+GEMOX** **54.5 (1.8)**	0.59 (0.18,1.94)	0.64 (0.18,2.30)	0.79 (0.23,2.72)	0.97 (0.28,3.44)	0.33 (0.07,1.62)	1.71 (0.49,5.97)	0.37 (0.09,1.63)	1.00 (0.26,3.87)	6.02 (0.27,132.13)	0.95 (0.18,4.97)
0.32 (0.10,1.03)	2.71 (0.12,60.16)	0.93 (0.03,28.60)	5.41 (0.22,130.85)	8.26 (0.32,214.12)	4.24 (0.15,117.74)	1.69 (0.52,5.56)	**Sorafenib** **30.7 (0)**	1.09 (0.69,1.72)	1.34 (0.95,1.88)	1.65 (1.08,2.51)	0.56 (0.20,1.61)	2.89 (1.96,4.26)	0.63 (0.26,1.51)	1.70 (0.89,3.22)	10.18 (0.59,176.22)	1.60 (0.50,5.09)
0.29 (0.08,1.03)	2.49 (0.11,57.18)	0.85 (0.03,27.10)	4.97 (0.20,124.24)	7.59 (0.28,203.16)	3.89 (0.14,111.64)	1.55 (0.43,5.57)	0.92 (0.58,1.46)	**Sunitinib** **36.2 (0)**	1.23 (0.69,2.18)	1.51 (0.81,2.83)	0.52 (0.16,1.63)	2.66 (1.45,4.85)	0.58 (0.22,1.55)	1.56 (0.71,3.43)	9.35 (0.52,167.96)	1.47 (0.42,5.11)
0.24 (0.07,0.81)	2.03 (0.09,45.91)	0.69 (0.02,21.79)	4.05 (0.16,99.80)	6.19 (0.23,163.25)	3.17 (0.11,89.73)	1.27 (0.37,4.37)	0.75 (0.53,1.06)	0.82 (0.46,1.45)	**Brivanib** **45.9 (0)**	1.23 (0.71,2.13)	0.42 (0.14,1.27)	2.16 (1.29,3.64)	0.47 (0.18,1.21)	1.27 (0.61,2.63)	7.62 (0.43,134.70)	1.20 (0.36,4.01)
0.19 (0.06,0.67)	1.65 (0.07,37.59)	0.56 (0.02,17.82)	3.28 (0.13,81.69)	5.02 (0.19,133.61)	2.57 (0.09,73.42)	1.03 (0.29,3.63)	0.61 (0.40,0.93)	0.66 (0.35,1.24)	0.81 (0.47,1.40)	**Linifanib** **56.2 (0.3)**	0.34 (0.11,1.06)	1.76 (0.99,3.12)	0.38 (0.15,1.01)	1.03 (0.48,2.22)	6.18 (0.35,110.38)	0.97 (0.28,3.33)
0.57 (0.12,2.74)	4.82 (0.18,127.19)	1.65 (0.05,59.51)	9.62 (0.34,275.38)	14.70 (0.48,449.13)	7.54 (0.23,246.14)	3.01 (0.62,14.71)	1.78 (0.62,5.08)	1.94 (0.62,6.09)	2.38 (0.79,7.17)	2.93 (0.94,9.08)	**Dovitinib** **16.5 (0)**	5.14 (1.68,15.74)	1.12 (0.29,4.39)	3.01 (0.88,10.32)	18.11 (0.87,377.86)	2.85 (0.60,13.58)
0.11 (0.03,0.38)	0.94 (0.04,21.32)	0.32 (0.01,10.11)	1.87 (0.08,46.34)	2.86 (0.11,75.79)	1.47 (0.05,41.65)	0.59 (0.17,2.05)	0.35 (0.23,0.51)	0.38 (0.21,0.69)	0.46 (0.28,0.78)	0.57 (0.32,1.01)	0.19 (0.06,0.60)	**Lenvatinib** **75.2 (2.3)**	0.22 (0.08,0.57)	0.59 (0.28,1.24)	3.52 (0.20,62.58)	0.55 (0.16,1.88)
0.51 (0.12,2.19)	4.30 (0.17,107.73)	1.47 (0.04,50.65)	8.58 (0.32,233.56)	13.12 (0.45,381.31)	6.73 (0.22,209.18)	2.69 (0.61,11.74)	1.59 (0.66,3.80)	1.73 (0.64,4.63)	2.12 (0.83,5.42)	2.61 (0.99,6.90)	0.89 (0.23,3.50)	4.59 (1.77,11.93)	**Nintedanib** **18.4 (0)**	2.69 (0.91,7.95)	16.17 (0.82,318.75)	2.54 (0.60,10.82)
0.19 (0.05,0.72)	1.60 (0.07,37.91)	0.55 (0.02,17.91)	3.19 (0.12,82.30)	4.87 (0.18,134.49)	2.50 (0.08,73.86)	1.00 (0.26,3.86)	0.59 (0.31,1.12)	0.64 (0.29,1.42)	0.79 (0.38,1.63)	0.97 (0.45,2.10)	0.33 (0.10,1.14)	1.71 (0.80,3.61)	0.37 (0.13,1.10)	**Sorafenib** **+Erlotinib** **56.2 (0.1)**	6.01 (0.32,111.65)	0.94 (0.25,3.55)
0.03 (0.00,0.68)	0.27 (0.00,17.96)	0.09 (0.00,7.87)	0.53 (0.01,38.19)	0.81 (0.01,61.43)	0.42 (0.01,33.21)	0.17 (0.01,3.65)	0.10 (0.01,1.70)	0.11 (0.01,1.92)	0.13 (0.01,2.32)	0.16 (0.01,2.89)	0.06 (0.00,1.15)	0.28 (0.02,5.04)	0.06 (0.00,1.22)	0.17 (0.01,3.09)	**Sorafenib+** **Everolimus** **84.4 (48.5)**	0.16 (0.01,3.41)
0.20 (0.04,1.04)	1.69 (0.06,46.30)	0.58 (0.02,21.60)	3.38 (0.11,100.17)	5.16 (0.16,163.26)	2.65 (0.08,89.41)	1.06 (0.20,5.55)	0.62 (0.20,1.99)	0.68 (0.20,2.36)	0.83 (0.25,2.79)	1.03 (0.30,3.53)	0.35 (0.07,1.67)	1.81 (0.53,6.12)	0.39 (0.09,1.68)	1.06 (0.28,3.98)	6.36 (0.29,137.94)	**Bevacizumab** **+Erlotinib** **52.2 (0.7)**

Values are risk ratio along with 95% CI. Value in diagonal line are SUCRAs, whereas the probability of being the best treatment is shown in parentheses.

FOLFOX4, fluorouracil, leucovorin, and oxaliplatin; GEMOX, gemcitabine, oxaliplatin; PIAF, cisplatin, interferon α-2b, doxorubicin, and fluorouracil.

### Adverse Events

Twenty RCTs (4–8, 18, 19, 21, 30–40) with 17 regimens were included to estimated RR (95% CI) of AEs see **Figure 3D** and **Table 8**. Most grade 3 or higher AEs in chemotherapy were neutropenia, whereas for MKIs, they were diarrhea or hand-foot skin reaction. All regimens except Nolatrexed, Dovitinib, Nintedanib, Sorafenib+Everolimus, and Bevacizumab+Erlotinib had significantly higher AEs compared to placebo, with RRs ranging from 4.99 to 33.83.

**Table 8:** Estimations of relative treatment effects of adverse events.

**Placebo** **97.1 (71.6)**	25.42 (1.40,459.86)	16.23 (0.83,317.01)	31.35 (1.55,632.38)	33.83 (1.65,693.19)	22.71 (1.09,474.23)	9.15 (2.08,40.22)	4.99 (1.85,13.48)	6.04 (1.67,21.81)	7.66 (2.11,27.81)	6.99 (1.92,25.49)	3.63 (0.82,16.16)	8.13 (2.23,29.68)	2.07 (0.55,7.81)	7.26 (1.98,26.59)	3.74 (0.81,17.14)	3.05 (0.53,17.36)
0.04 (0.00,0.71)	**Doxorubicin** **24.7 (0)**	0.64 (0.33,1.25)	1.23 (0.55,2.75)	1.33 (0.56,3.14)	0.89 (0.36,2.25)	0.36 (0.01,9.31)	0.20 (0.01,4.19)	0.24 (0.01,5.64)	0.30 (0.01,7.17)	0.28 (0.01,6.56)	0.14 (0.01,3.71)	0.32 (0.01,7.63)	0.08 (0.00,1.97)	0.29 (0.01,6.83)	0.15 (0.01,3.87)	0.12 (0.00,3.51)
0.06 (0.00,1.20)	1.57 (0.80,3.06)	**Nolatrexed** **39.5 (2.8)**	1.93 (0.68,5.49)	2.08 (0.70,6.19)	1.40 (0.45,4.37)	0.56 (0.02,15.72)	0.31 (0.01,7.12)	0.37 (0.01,9.55)	0.47 (0.02,12.14)	0.43 (0.02,11.10)	0.22 (0.01,6.27)	0.50 (0.02,12.91)	0.13 (0.00,3.34)	0.45 (0.02,11.55)	0.23 (0.01,6.54)	0.19 (0.01,5.92)
0.03 (0.00,0.64)	0.81 (0.36,1.81)	0.52 (0.18,1.47)	**PIAF** **19.1 (0)**	1.08 (0.33,3.49)	0.72 (0.21,2.46)	0.29 (0.01,8.32)	0.16 (0.01,3.77)	0.19 (0.01,5.05)	0.24 (0.01,6.43)	0.22 (0.01,5.88)	0.12 (0.00,3.32)	0.26 (0.01,6.84)	0.07 (0.00,1.76)	0.23 (0.01,6.11)	0.12 (0.00,3.46)	0.10 (0.00,3.13)
0.03 (0.00,0.61)	0.75 (0.32,1.77)	0.48 (0.16,1.42)	0.93 (0.29,3.00)	**FOLFOX4** **17.0 (0)**	0.67 (0.19,2.36)	0.27 (0.01,7.81)	0.15 (0.01,3.55)	0.18 (0.01,4.75)	0.23 (0.01,6.04)	0.21 (0.01,5.52)	0.11 (0.00,3.12)	0.24 (0.01,6.42)	0.06 (0.00,1.66)	0.21 (0.01,5.75)	0.11 (0.00,3.25)	0.09 (0.00,2.94)
0.04 (0.00,0.92)	1.12 (0.45,2.81)	0.71 (0.23,2.23)	1.38 (0.41,4.68)	1.49 (0.42,5.25)	**Sorafenib+** **Doxorubicin** **28.8 (1.1)**	0.40 (0.01,11.84)	0.22 (0.01,5.38)	0.27 (0.01,7.20)	0.34 (0.01,9.16)	0.31 (0.01,8.37)	0.16 (0.01,4.72)	0.36 (0.01,9.74)	0.09 (0.00,2.51)	0.32 (0.01,8.71)	0.16 (0.01,4.92)	0.13 (0.00,4.45)
0.11 (0.02,0.48)	2.78 (0.11,71.76)	1.77 (0.06,49.43)	3.42 (0.12,97.54)	3.70 (0.13,106.75)	2.48 (0.08,72.89)	**Sorafenib** **+GEMOX** **36.6 (0)**	0.55 (0.18,1.63)	0.66 (0.17,2.59)	0.84 (0.21,3.30)	0.76 (0.19,3.02)	0.40 (0.08,1.90)	0.89 (0.22,3.52)	0.23 (0.06,0.92)	0.79 (0.20,3.15)	0.41 (0.08,2.01)	0.33 (0.05,2.02)
0.20 (0.07,0.54)	5.09 (0.24,108.71)	3.25 (0.14,75.23)	6.28 (0.27,148.65)	6.78 (0.28,162.82)	4.55 (0.19,111.28)	1.83 (0.61,5.49)	**Sorafenib** **61.7 (0)**	1.21 (0.54,2.73)	1.53 (0.68,3.49)	1.40 (0.61,3.21)	0.73 (0.24,2.22)	1.63 (0.71,3.74)	0.41 (0.17,1.00)	1.46 (0.63,3.35)	0.75 (0.24,2.38)	0.61 (0.15,2.55)
0.17 (0.05,0.60)	4.21 (0.18,99.94)	2.69 (0.10,68.98)	5.19 (0.20,136.20)	5.60 (0.21,149.11)	3.76 (0.14,101.86)	1.52 (0.39,5.94)	0.83 (0.37,1.86)	**Sunitinib** **52.2 (0.1)**	1.27 (0.40,4.03)	1.16 (0.36,3.70)	0.60 (0.15,2.39)	1.35 (0.42,4.30)	0.34 (0.10,1.13)	1.20 (0.38,3.86)	0.62 (0.15,2.54)	0.50 (0.10,2.61)
0.13 (0.04,0.47)	3.32 (0.14,78.93)	2.12 (0.08,54.47)	4.09 (0.16,107.55)	4.42 (0.17,117.75)	2.96 (0.11,80.43)	1.19 (0.30,4.70)	0.65 (0.29,1.48)	0.79 (0.25,2.50)	**Brivanib** **43.1 (0.1)**	0.91 (0.28,2.93)	0.47 (0.12,1.89)	1.06 (0.33,3.41)	0.27 (0.08,0.90)	0.95 (0.29,3.06)	0.49 (0.12,2.01)	0.40 (0.08,2.07)
0.14 (0.04,0.52)	3.63 (0.15,86.62)	2.32 (0.09,59.78)	4.48 (0.17,118.03)	4.84 (0.18,129.22)	3.25 (0.12,88.27)	1.31 (0.33,5.17)	0.71 (0.31,1.63)	0.86 (0.27,2.76)	1.10 (0.34,3.52)	**Linifanib** **45.6 (0.1)**	0.52 (0.13,2.08)	1.16 (0.36,3.75)	0.30 (0.09,0.99)	1.04 (0.32,3.36)	0.53 (0.13,2.21)	0.44 (0.08,2.27)
0.28 (0.06,1.23)	7.00 (0.27,181.91)	4.47 (0.16,125.28)	8.63 (0.30,247.24)	9.32 (0.32,270.58)	6.25 (0.21,184.75)	2.52 (0.53,12.04)	1.37 (0.45,4.19)	1.66 (0.42,6.61)	2.11 (0.53,8.42)	1.93 (0.48,7.72)	**Dovitinib** **70.7 (2.2)**	2.24 (0.56,8.99)	0.57 (0.14,2.35)	2.00 (0.50,8.05)	1.03 (0.21,5.12)	0.84 (0.14,5.14)
0.12 (0.03,0.45)	3.13 (0.13,74.54)	2.00 (0.08,51.44)	3.85 (0.15,101.57)	4.16 (0.16,111.19)	2.79 (0.10,75.96)	1.13 (0.28,4.45)	0.61 (0.27,1.41)	0.74 (0.23,2.37)	0.94 (0.29,3.03)	0.86 (0.27,2.78)	0.45 (0.11,1.79)	**Lenvatinib** **40.1 (0.1)**	0.25 (0.08,0.85)	0.89 (0.28,2.90)	0.46 (0.11,1.90)	0.37 (0.07,1.96)
0.48 (0.13,1.83)	12.29 (0.51,297.32)	7.85 (0.30,205.52)	15.16 (0.57,404.96)	16.36 (0.60,443.31)	10.98 (0.40,302.80)	4.43 (1.09,18.03)	2.41 (1.00,5.80)	2.92 (0.88,9.66)	3.71 (1.11,12.32)	3.38 (1.01,11.29)	1.76 (0.43,7.25)	3.93 (1.18,13.15)	**Nintedanib** **86.8 (10.7)**	3.51 (1.05,11.79)	1.81 (0.42,7.70)	1.47 (0.28,7.88)
0.14 (0.04,0.50)	3.50 (0.15,83.54)	2.23 (0.09,57.65)	4.31 (0.16,113.82)	4.66 (0.17,124.61)	3.13 (0.11,85.12)	1.26 (0.32,5.00)	0.69 (0.30,1.58)	0.83 (0.26,2.67)	1.05 (0.33,3.40)	0.96 (0.30,3.12)	0.50 (0.12,2.01)	1.12 (0.35,3.63)	0.28 (0.08,0.95)	**Sorafenib** **+Erlotinib** **44.9 (0)**	0.51 (0.12,2.14)	0.42 (0.08,2.19)
0.27 (0.06,1.23)	6.80 (0.26,179.38)	4.35 (0.15,123.50)	8.39 (0.29,243.70)	9.06 (0.31,266.69)	6.08 (0.20,182.08)	2.45 (0.50,12.05)	1.34 (0.42,4.24)	1.62 (0.39,6.64)	2.05 (0.50,8.46)	1.87 (0.45,7.75)	0.97 (0.20,4.84)	2.18 (0.52,9.03)	0.55 (0.13,2.36)	1.94 (0.47,8.09)	**Sorafenib** **+Everolimus** **68.4 (2.2)**	0.82 (0.13,5.12)
0.33 (0.06,1.87)	8.35 (0.28,244.81)	5.33 (0.17,168.16)	10.29 (0.32,331.60)	11.11 (0.34,362.73)	7.46 (0.22,247.53)	3.01 (0.50,18.22)	1.64 (0.39,6.85)	1.98 (0.38,10.27)	2.52 (0.48,13.09)	2.30 (0.44,11.98)	1.19 (0.19,7.30)	2.67 (0.51,13.95)	0.68 (0.13,3.63)	2.39 (0.46,12.49)	1.23 (0.20,7.71)	**Bevacizumab** **+Erlotinib** **73.9 (9.0)**

Values are risk ratio along with 95% CI. Value in diagonal line are SUCRAs, whereas the probability of being the best treatment is shown in parentheses.

FOLFOX4, fluorouracil, leucovorin, and oxaliplatin; GEMOX, gemcitabine, oxaliplatin; PIAF, cisplatin, interferon α-2b, doxorubicin, and fluorouracil.

There was no significant difference in AEs among chemotherapy and combined MKI regimens. Only Nintedanib had significantly lower AEs than Sorafenib+GEMOX, Lenvatinib, Brivanib, and Linifanib with RRs of 0.23 (0.06, 0.92), 0.25 (0.08, 0.85), 0.27 (0.08, 0.90), and 0.30 (0.09, 0.99), respectively. The best ranked regimens in terms of having the least AEs were Nintedanib and Bevacizumab+Erlotinib, see **Table 8**.

### Risk and Benefit Assessment

**Figure 4:**
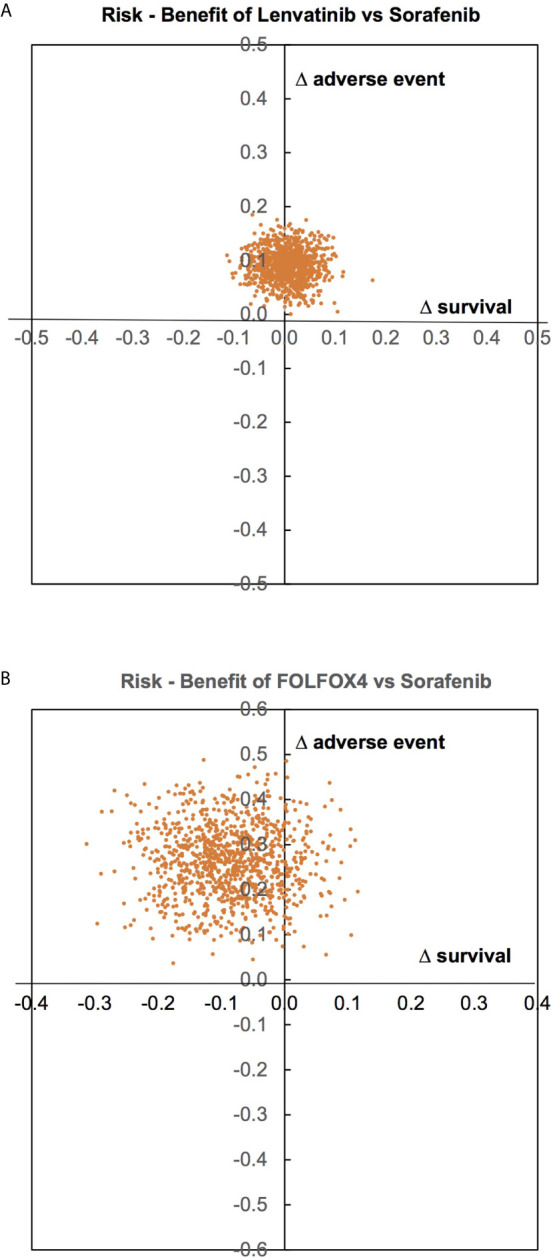
Risk of adverse events and benefit of overall survival assessment. (**A** – between Lenvatinib and Sorafenib, **B** – between FOLFOX4 and Sorafenib).

Risk (AEs) and benefit (OS) were assessed by estimating the incremental risk-benefit ratio of the treatments recommended by clinical practice guidelines, i.e., Sorafenib, Lenvatinib, and FOLFOX4. Incremental risk-benefit ratios of Lenvatinib and FOLFOX4 versus Sorafenib were jointly simulated for each pair using 1000 Monte Carlo simulations, and then risk of AEs and benefit of OS were plotted on y-and x-axis, respectively, see **Figure 4**.

For Lenvatinib versus Sorafenib, most AEs and OS fell in the right and the left upper quadrants with symmetry about the x-axis, but not in the y-axis see **Figure 4A**; indicating that Lenvatinib offered no benefit in terms of OS, but had higher AEs compared to Sorafenib. FOLFOX4 appears to have lower OS and higher AEs than Sorafenib see **Figure 4B**.

## Discussion

We conducted a NMA to assess the efficacy of first-line HCC treatments focusing on chemotherapy agents, and MKIs using data from 20 phase II-III RCTs. Our findings suggested that Lenvatinib and FOLFOX4 were the best of the MKIs and chemotherapy agents respectively, prolonging OS and PFS by 9 and 6.3 months and 7.9 and 4.3 months respectively without significantly different AEs relative to other agents in their class. Combining these classes, i.e. Sorafenib+Doxorubicin could prolong OS and PFS to as long as 12.7 and 6.3 months.

Our NMA included all chemotherapy agents and MKIs which had been studied in RCTs up to 2019, including Lenvatinib (8), which was not included in the previous NMA (9). This allowed us to compare the efficacy between chemotherapy and MKIs by borrowing common comparators (e.g., placebo, Sorafenib), which also had not been done before. The previous NMA (9) indicated that Sorafenib, Linifanib, Brivanib, and Sunitinib were significantly better in prolonging OS than placebo, and that the combination of Sorafenib+Erlotinib was the best in prolonging OS, followed by Sorafenib. Our updated findings indicated that Lenvatinib is better than Sorafenib in prolonging OS, PFS, and showed higher ORR. Considering only treatment regimens recommended by the National Comprehensive Cancer Network (NCCN) (41), including Sorafenib, Lenvatinib, and FOLFOX4, our findings showed that Lenvatinib provides the longest OS and PFS with no significant difference in AEs compared to other treatments.

For chemotherapy agents, only direct meta-analysis of Oxaliplatin-based chemotherapy had been published (12, 42). The pooled median PFS for Oxaliplatin-based chemotherapy was similar to our FOLFOX4 regimen (4.2 (12) to 4.7 (42) months vs. 4.3 months) but OS was slightly longer than our results (9.3 (12) to 9.4 (42) months vs 7.9 months). The difference in OS may come from heterogeneity in the combination of chemotherapy with Oxaliplatin.

Although our results favored MKIs as the treatment of choice in advanced HCC, many developing countries may have less access to these drugs due to high cost factors. Therefore, FOLFOX4 may still be considered as the best treatment option in these settings, as suggested by our findings. FOLFOX4 was ranked the highest among chemotherapy agents, but still showed shorter OS and higher AEs rates compared to Sorafenib and Lenvatinib.

The combination of chemotherapy and MKIs (i.e., Sorafenib+Doxorubicin or Sorafenib+GEMOX) or the combination of MKIs (Sorafenib+Erlotinib, Sorafenib+Everolimus, or Bevacizumab+Erlotinib) may also hold some promising outcome for improving OS and PFS. However, all of the combination treatments except Sorafenib+Erlotinib were from phase II RCTs (21, 30, 31, 38), which may overestimate the efficacy due to high selection bias in those trials. Those findings require further evidence for confirmation.

Our study has some strengths. Firstly, we extracted time along with probability of OS/PFS from Kaplan-Meier curves using Digitizer software. Numbers of events (i.e., death and disease progression) along with person-time at each distinct point of the Kaplan-Meier curve were extracted. These data were then converted to individual patient data using methods suggested by Wei and Royston (26). This allowed us to be more flexible in applying a mixed-effect model (28) for time to event data analysis. Median OS and PFS were then estimated for each regimen. Among 20 RCTs, five studies did not report numbers of events and person-time at risk at each distinct time. Therefore, the estimation of hazard functions for these studies might be biased as their hazard ratios, which were estimated from individual level data, were different from their actual reported data.

Nonetheless, there were some limitations, as our review included primary studies published from 1988 to 2019. Since studies of MKIs are more recent than that of chemotherapy, their longer survival times may be confounded with improvements in supportive care over time. Recently, combination immune checkpoint inhibitor (Atezolizumab+Bevacizumab) (43) has been approved as the first-line treatment of advanced HCC and newer treatment options are still being studied in clinical trials. However, accessibility to these treatments is still limited particularly in developing countries due to their costs. Due to the high cost of treatment and grave prognosis of the disease, preventive strategy should be promoted and applied to decrease the incidence of new cases. Adjunctive treatments, which could modify tumorigenesis pathways or major risk factors of HCC such as viral hepatitis B/C infections with epigenetics, microRNAs or microenvironment modification (44, 45), should be explored in clinical studies to seek novel therapeutic options for improving survival and quality of life for HCC patients.

In conclusion, for limited resource countries, results from this NMA support the use of MKIs, i.e., Lenvatinib or Sorafenib, as a first-line systemic treatment for advanced HCC with preserved liver function (Child-Pugh A) and non-significant portal vein involvement. FOLFOX4, chemotherapy, might be an option if MKIs are less accessible. This NMA should be updated when more phase-III RCTs are published.

## Data Availability Statement

The original contributions presented in the study are included in the article/supplementary material. Further inquiries can be directed to the corresponding author.

## Author Contributions

SO: Conceptualization, Methodology, Formal analysis, Investigation, Writing-Original draft preparation. AP and ATa: Validation. SR and TR: Supervision. ATh: Writing-Review and editing, Supervision. JA: Writing-Review and editing. All authors contributed to the article and approved the submitted version.

## Conflict of Interest

The authors declare that the research was conducted in the absence of any commercial or financial relationships that could be construed as a potential conflict of interest.

## Abbreviation

AE, adverse event; AIC, Akaike’s information criterion; CI, confidence interval; FDA, Food and Drug Administration; FOLFOX4, Fluorouracil, Leucovorin, and Oxaliplatin; GEMOX, Gemcitabine and Oxaliplatin; HCC, hepatocellular carcinoma; MKIs, multikinase inhibitors; NCCN, the National Comprehensive Cancer Network; NMA, network meta-analysis; ORR, overall response rate; OS, overall survival; PFS, progression-free survival; PIAF, Cisplatin, Interferon α-2b, Doxorubicin, and Fluorouracil; PRISMA, Preferred Items for Systematic Reviews and Meta-Analysis; RCT, randomised-controlled trial; RECIST, the response evaluation criteria in solid tumors; RoB 2, the Revised Cochrane Risk of Bias tool; RR, risk ratio; SR, systematic review; SUCRA, surface under the cumulative ranking curve; WHO, World Health Organization.
